# Avacopan improves patient perspective on steroid-related toxicity effects in a case with ANCA-associated vasculitis

**DOI:** 10.1093/rap/rkad058

**Published:** 2023-07-05

**Authors:** Peter Korsten, Björn Tampe

**Affiliations:** Department of Nephrology and Rheumatology, University Medical Center Göttingen, Göttingen, Germany; Department of Nephrology and Rheumatology, University Medical Center Göttingen, Göttingen, Germany

Key message

- Our report describes the patient perspective added value of avacopan in AAV treatment, and that beneficial effects of avacopan in the absence of steroid-related side events are also observed from a patient’s perspective.

Dear Editor, It has recently been shown that the compassionate use of avacopan in difficult-to-treat ANCA-associated vasculitis (AAV) is capable of reducing clinical steroid-related toxicity effects (BMI, glucose tolerance, blood pressure or lipid metabolism) [1]. For the first time, we have systematically assessed patient perspective on steroid-related toxicity effects by established side effects rating and report that avacopan improves steroid-related toxicity effects in a case with ANCA-associated renal vasculitis [2].

A 74-year-old woman with pre-existing AAV presented to our tertiary centre for an ambulatory control visit. AAV was first diagnosed 5 years ago, with pulmonary involvement and biopsy-confirmed ANCA-associated renal vasculitis. For remission induction therapy, the patient was treated with steroids and CYC and received steroids and AZA for maintenance therapy. The patient developed a major relapse, with biopsy-confirmed crescentic ANCA-associated renal vasculitis and received steroids and rituximab for remission induction 3 months before the current visit. At presentation, the patient was tapered down to 30 mg of oral prednisone and reported progressive weakness, peripheral oedema of the lower legs and worsening of insulin-dependent diabetes. Assessment of the patient perspective on steroid-related toxicity effects confirmed predominant mood changes, diabetes, high blood pressure, indigestion, reduced bone strength, round face and weight gain to be predominant side effects (Fig. 1) [2]. Therefore, avacopan treatment (30 mg twice daily) was initiated and prednisone gradually tapered down. At follow-up visits 3 and 6 weeks thereafter, the rating of patient perspective on steroid-related toxicity effects gradually decreased for most side effects (Fig. 1).

**Figure 1.**
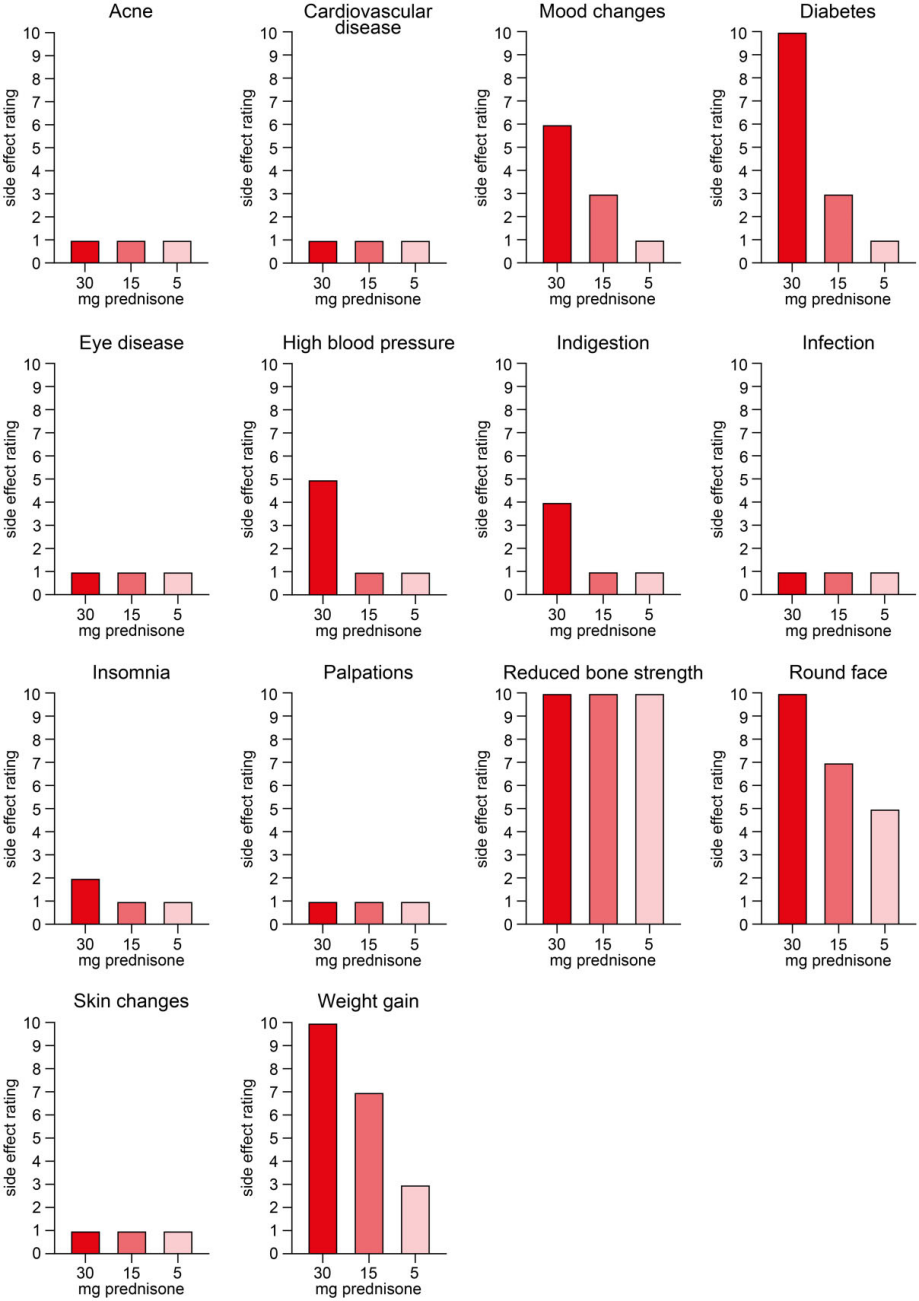
Assessment of patient perspectives on steroid-related toxicity effects. Patient perspectives on steroid-related toxicity effects were scored at presentation (30 mg oral prednisone), 3 weeks (15 mg oral prednisone) and 6 weeks (5 mg oral prednisone) thereafter

In summary, we report here that avacopan improved the patient perspective on steroid-related toxicity effects in a case with AAV. The challenge to identify beneficial effects of avacopan in clinical data of patients with AAV is defined by the absence of disease activity, disease flares, steroids and steroid-related toxicity. It requires careful consideration to determine benefit firmly by proving the absence of clinically relevant events. Furthermore, it will remain a significant challenge to prove the clinical efficacy of avacopan on the background of highly intensive immunosuppression necessary for remission induction in AAV.

For clinical evaluation, the Glucocorticoid Toxicity Index (GTI) to assess glucocorticoid-related morbidity and glucocorticoid-sparing ability of other therapies has been described [3]. However, the patient perspective on steroid-related toxicity effects is also of relevance, especially for individual quality of life and adherence to therapy. Clinicians and patients make treatment decisions after weighing the benefits against the possible harms, considering their probability, nature and a value judgement of how important it is to the individual [4]. Although many studies have estimated the frequency of side effects, few have considered how important they are to individual patients [5–8]. This is especially relevant, because the patient perspective about a given side effect will influence the decisions about treatment and adherence. Recently, an online cross-sectional survey identified that weight gain, insomnia and moon face were the top three most important steroid-related side effects from the patient perspective. These are not clinically serious but remain important to patients, perhaps reflecting their impact on quality of life and high prevalence [2]. This should be taken into consideration when discussing treatment options and planning future steroid safety studies. Thus, we emphasize the assessment of clinical and patient perspectives on steroid-related toxicity effects using validated scoring systems and disease-relevant patient-reported outcomes. Alternative use of avacopan in cases with steroid-related side effects could be beneficial in difficult-to-treat patients with AAV. Our report describes the patient perspective added value of avacopan in AAV treatment, and that beneficial effects of avacopan in the absence of steroid-related side events are also observed from a patient’s perspective.

## Data Availability

All data are incorporated into the article.
